# A Single-Center Clinical Experience with Fully Percutaneous, Minimally Invasive Fetoscopic Surgery for Spina Bifida Aperta

**DOI:** 10.3390/biomedicines13112625

**Published:** 2025-10-27

**Authors:** Robert Brawura Biskupski Samaha, Mirosław Wielgoś, Thomas Kohl, Michal Lipa, Ksawery Goławski, Katarzyna Kosińska-Kaczyńska, Katarzyna Luterek, Przemysław Kosiński, Julia Sienczyk

**Affiliations:** 1 Department of Obstetrics, Perinatology and Neonatology, Center of Postgraduate Medical Education, 01-809 Warsaw, Poland; katarzyna.kosinska-kaczynska@cmkp.edu.pl; 2 Medical Faculty, Lazarski University, 02-662 Warsaw, Poland; miroslaw.wielgos@pimmswia.gov.pl; 3 National Medical Institute of the Ministry of the Interior and Administration in Warsaw, 02-507 Warszawa, Poland; michal.lipa@pimmswia.gov.pl (M.L.); ksawery.golawski@pimmswia.gov.pl (K.G.); 4 Deutsches Zentrum für Fetalchirurgie & Minimal-Invasive Therapie (DZFT), University Hospital Mannheim (UMM), 68167 Mannheim, Germany; thomas.khol@umm.de; 5 1st Department of Obstetrics and Gynecology, Warsaw Medical University, 02-091 Warsaw, Poland; katarzyna.luterek@wum.edu.pl; 6 Department of Obstetrics, Perinatology, Gynecology and Reproductive Medicine, Medical University of Warsaw, 02-091 Warsaw, Poland; pkosinski@wum.edu.pl; 7 Medical Faculty, Medical University of Warsaw, 02-091 Warsaw, Poland; julasienczyk@gmail.com

**Keywords:** fetoscopy, spina bifida, fetoscopic surgery, early clinical experience

## Abstract

**Background/Objectives**: Following a tailored curriculum, minimally invasive fetoscopic coverage for spina bifida aperta (SBA) was introduced in Poland in 2017. This study aims to present the results of the first patients that underwent this procedure in the 1st Department of Obstetrics and Gynecology, Medical University of Warsaw and compare them with the results obtained in other studies. **Methods**: We reviewed our data of 38 expectant mothers whose fetuses with SBA and normal karyotype underwent minimally invasive fetoscopic coverage at our center between September 2017 and February 2022. All procedures were carried out between 24 + 4 and 28 + 1 weeks of gestation employing general materno-fetal anesthesia. New methods were implemented with time, moving from the patch technique to the skin-to-skin technique suture. The results of the study were compared with the available literature on fetoscopic and open surgeries. **Results**: In total, the procedure was attempted 38 times and completed in 34 cases. All lesions were lumbar, and the median width of the lateral ventricle was 12 mm (6–17 mm). The median age at surgery was 26 weeks and the median age at delivery was 32 weeks of gestation (26.1–37.5). The average birth weight was 1870 g (1070–3350g). From 34 patients to 31 at the one year follow-up, 13 out of 31 (41.9%) babies needed a shunt and more than 70% of babies had a functional motor level that was the same or better than the anatomical level. **Conclusions**: Minimally invasive surgery for SBA could successfully be implemented following a tailored curriculum at our university with encouraging maternal and neonatal outcomes. The fetoscopic approach permits the assessment of various closure approaches. Preterm delivery is common but usually occurs beyond 30 weeks of gestation. At this time relevant complications from prematurity are rare.

## 1. Introduction

The multicenter randomized controlled trial Management of Myelomeningocele Study (MOMS) and other trials demonstrated that prenatal repair of spina bifida aperta (SBA) better preserves the motor and sensory function of affected fetuses, helps reverse hindbrain herniation and lowers the need for hydrocephalus treatment as compared to the postnatal repair [1,2,3,4,5,6,7,8]. However, as this approach is by hysterotomy, it carries a risk of uterine dehiscence and rupture in the index and subsequent pregnancies. To minimize maternal morbidity from open fetal surgery, a fully percutaneous minimally invasive fetoscopic approach for fetal SBA closure was developed more than two decades ago by pediatrician Thomas Kohl [3,9,10,11]. Similarly to the open operative approach, lower rates of shunt insertions for hydrocephalus and better-preserved leg function have been observed postnatally after this procedure [4].

Minimally invasive surgeries do not cause uterine dehiscence but have a higher rate of premature rupture of membranes (PROM) and chorionic membrane separation [8,12]. When comparing the two fetoscopic approaches, totally percutaneous vs. laparotomy-assisted, the main advantage of the latter is a lower rate of PROM and a higher gestational age at delivery [8,12]. It is noteworthy that Chmait et al. introduced a new interesting technique called percutaneous/mini-laparotomy fetoscopy, in which access to the uterus for one of the ports is performed via a mini-laparotomy, whereas the other ports are inserted percutaneously [13].

Open fetal surgery, involving laparotomy and hysterotomy has been performed in Poland since 2005 at the Medical University of Silesia in Bytom [14]. However, an alternative, less invasive fetoscopic approach has not been performed and analyzed. Therefore, there is a need to evaluate the preliminary results of the first operations of this type of surgery performed in Poland.

This study aims to present our early clinical experience, main obstetrical, perinatal, and neurosurgical outcomes up to 12 months of age and to compare our results with those from other groups performing fetoscopic and open neural tube defect repair.

## 2. Materials and Methods

### 2.1. Study Design

This retrospective cohort study included all consecutive fetoscopic SBA repair procedures at our center. The report of this study was structured and written following the STROBE (Strengthening the Reporting of Observational Studies in Epidemiology) guidelines to ensure accurate and standardized reporting of the observational data [15].

### 2.2. Setting

This study was conducted at the 1st Department of Obstetrics and Gynecology of the Medical University of Warsaw, Poland. This site is distinguished as the inaugural center in Poland to implement the procedure under investigation. The study timeframe spans from September 2017 to May 2022. This period was selected based on the initial adoption of the procedure at this facility, allowing for a comprehensive evaluation of outcomes post-intervention.

The initiation of fetoscopic fetal surgery for SBA in Poland was preceded by an extensive training regimen undertaken by a multidisciplinary team consisting of fetal medicine specialists, anesthesiologists, and nursing staff. This training was conducted at the German Center for Fetal Surgery & Minimally Invasive Surgery (DZFT). From June 2016 to August 2017, the team participated in a series of 11 visits to the DZFT, which included observing live surgeries and engaging in both theoretical and hands-on instruction. The first three procedures performed in Poland were under the supervision and with the assistance of Professor Thomas Kohl.

### 2.3. Participants

Expectant mothers carrying fetuses affected with spina bifida were referred after their 20-week anomaly scan to our center by their local obstetrician or prenatal medicine specialist.

Following a detailed ultrasound scan at our unit, they were counseled about the malformation and possible clinical courses. Counseling included the options of conservative management followed by standard postnatal surgery, as well as the possibility of open or totally percutaneous fetoscopic surgery. In addition, all patients had an amniocentesis and karyotyping of the fetus. Prior to the procedures, all women underwent physical and laboratory evaluations, repeated fetal sonography, as well as transvaginal assessment of their uterine cervical competence. Magnetic resonance imaging was not required but was performed in some cases.

The inclusion and exclusion criteria are listed in Table 1. As maternal safety was our primary concern, women with conditions that would disproportionately increase their anesthetic or surgical risks were to be excluded. Some of the maternal exclusion criteria are: HELLP (hemolysis, elevated liver enzymes, low platelets), PE (preeclampsia), PPROM (preterm premature rupture of membranes), cardiomyopathy or a complex congenital heart disease, mechanical valves, reduced systemic left ventricular function, Marfan’s syndrome (with or without aortic dilatation), and pulmonary hypertension as well as women with a high risk of thrombosis (such as a homozygous factor V Leiden mutation, homozygous prothrombin gene mutation, or protein C or S deficiency with a family history of deep vein thrombosis). Patients with an anterior placenta and/or very thick abdominal walls were informed that the procedure would carry a somewhat higher risk for being technically unsuccessful. In addition, we adhered to previously published criteria for fetal selection [2,4]. If no maternal or fetal exclusion criteria were found, informed consent was obtained.

### 2.4. Procedures

#### 2.4.1. Maternal–Fetal Anesthesia and Intraoperative Monitoring

All procedures were carried out between 24 + 4 and 28 + 1 weeks of gestation employing general maternal-fetal anesthesia following a dedicated protocol [16,17]. Rapid sequence induction of anesthesia was performed using IV remifentanil (1 mg/kg), thiopental (5 mg/kg), cisatracurium (0.015 mg/kg), and succinylcholine (1 mg/kg). Remifentanil infusion and concentration of desflurane were adjusted to maternal and fetal requirements [16,17,18].

Intra- and post-operative maternal monitoring included electrocardiography, systemic arterial and central venous blood pressures, oxygen saturation, urine production, bispectral index, extravascular lung water, and body temperature.

#### 2.4.2. Fully Percutaneous Minimally Invasive Fetoscopic Approach

**Figure 1:**
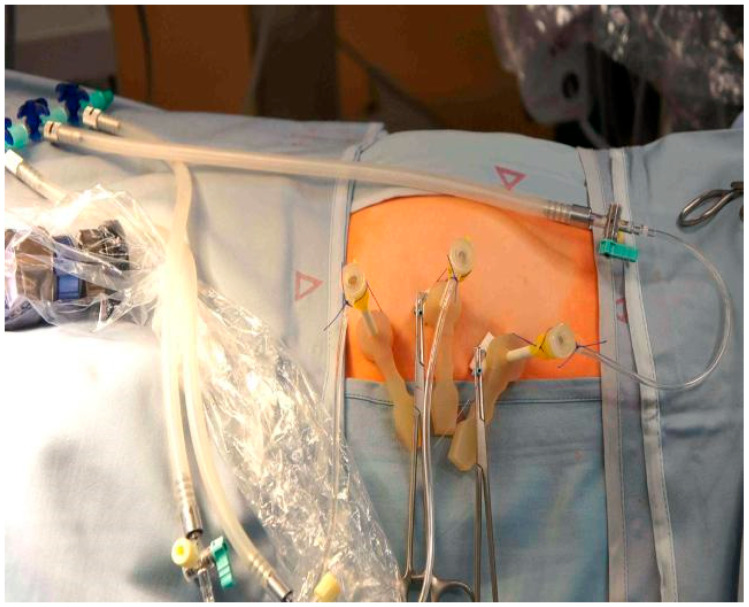
Fully percutaneous minimally invasive fetoscope for spina bifida. Three trocars introduced to uterine cavity.

Under maternal transabdominal ultrasound guidance, three trocars (external trocar diameter 5 mm—Terumo Radifocus Introducer II R*SA 11K10SQ Fr. 11) (Miniature Straight Forward Telescope, Karl Stortz, Tuttlingen, Germany) were percutaneously inserted into the amniotic cavity using the Seldinger technique (Figure 1) [9]. However, the first needle we use to enter the amniotic cavity is the Entuit^®^ Secure Adjustable Gastrointestinal Suture Anchor Set. Thus, the hollow needle that would be used in the Seldinger technique is substituted with a needle holding the anchor set. After entering the uterus, we introduce the round-tipped guide wire that pushes the anchor into the amniotic cavity. This anchor is attached to a string outside the abdominal wall. Wrenching the string we pull back the anchor against the amniotic sac and uterine wall, holding the two together and somewhat pulling the uterus towards the abdominal wall of the patient. We then continue with introducing the trocar. The anchor stays inside the amniotic cavity holding the amniotic sac against the uterus. In three cases where T-fasteners were not available as a means for maintaining apposition of membranes, uterine wall, and maternal abdominal wall throughout the procedure, we placed anti-eventration sutures close to the trocar sites.

Trocar insertion was followed by partial evacuation of amniotic fluid and carbon dioxide insufflation of the amniotic cavity. This strategy improves fetal visualization and surgical manipulation [19]. Humidification and heating of the CO_2_ (Lexion Medical, Minneapolis, MN, USA) was used in the second half of the cases.

When the fetal proper position was achieved with clear access to the back of the fetus, the lesion was circumcised at the border with healthy skin with a needle electrode, then the neural tissue was carefully freed from surrounding tissues. In the first cases, these steps were followed by suturing of a collagen patch covering the area of the lesion.

Various closure techniques were assessed over time, among them patch closure (Cook Medical Biodesign 4-layer tissue graft—REF SLH–4S–7X10-2 or Nevelia Bi-layer matrix MCS0505), direct closure over a biocellulose patch (Gore, Preclude Pericardial Membrane. Catalog number 1PCM001), and multilayer closure with an additional myofascial flap. The techniques used evolved due to the team’s constant training and exchange of experiences with other surgeons performing this procedure worldwide.

As in our series, most of the lesions were small; we soon began to abandon the sole use of patches. Instead, following the dissection of the placode, we attempted to close the skin directly over a biocellulose patch as had been described by Professor Denise Lapa [20].

To bring together the two edges of the wound, we mobilized the whole defect area by bluntly separating the skin from the subcutaneous tissue with the use of scissors. After we visited Lapa’s team in Sao Paulo, Brazil, we switched from four running sutures for patch closure to a single running suture with longer jumps between each stitch. We also added a myofascial flap to the procedure with the aim of achieving a more watertight closure [7,21,22]. We forewent the use of lateral skin incisions for large lesions. Whenever a malformation was too large for a skin-to-skin closure, i.e., in cases where we were unable to bring the skin edges together, we chose patch coverage (Table 2).

After fetoscopic coverage of the defect, the insufflation was halted, the CO_2_ evacuated, and the amniotic cavity refilled with warmed Ringer’s solution. Then, the trocars were removed and the abdominal trocar insertion sites sutured by single stitches. After these steps, mother and fetus were recovered from anesthesia.

#### 2.4.3. Perioperative and Delivery Management

Perioperative antibiotic prophylaxis, starting one hour before surgery, was performed with Clindamycin (4 × 600 mg) and Gentamycin (2 × 120 mg) until the third postoperative day. In cases of early postoperative amniotic fluid leakage, the antibiotics were continued. Tocograms were registered twice daily. Postoperative tocolysis was performed by infusion of the oxytocin antagonist atosiban for 24 h. Following minimally invasive fetoscopic closure for SBA, fetal delivery was planned after completion of 37 weeks of gestation. In cases with amniotic fluid leakage before 34 weeks, prophylactic antibiotics were given, and delivery was scheduled at completion of 34 weeks.

#### 2.4.4. Variables and Measurement

The following parameters were assessed: total number of procedures performed and completed, mean maternal age and body mass index, type and level of lesion, gestational age at surgery and delivery, skin-to-skin time, fetoscopic repair time, insufflation time, and maternal and fetal complications. In addition, data about the early postnatal survival, need for shunt insertion, and anatomical-to-functional level were described.

### 2.5. Statistical Methods

The obtained results were compared with available data published by other researchers. The analysis was performed using the R language in the RStudio (version 4.4) environment. For the comparison of quantitative variables, T-tests were utilized, whereas qualitative variables were analyzed using the Chi-squared test. In cases where full data regarding standard deviation were not available, the T-test for a single sample was employed, using the available value as the population mean. Values of *p* < 0.05 were considered significant.

### 2.6. Ethical Consideration

All procedures were performed in accordance with the Helsinki declaration and according to institutional guidelines for patient protection. The study was approved by our local Bioethical Committee of the Medical University of Warsaw (KB/22/2021).

## 3. Results

**Figure 2:**
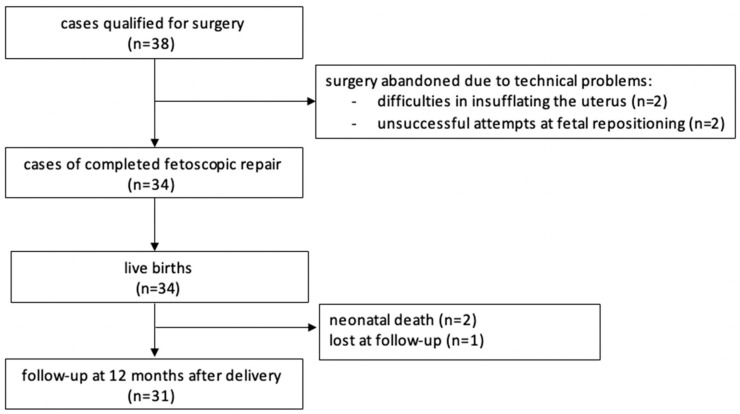
Flow chart.

In total, the procedure was attempted 38 times and completed in 34 cases. One patient was lost to follow-up (Figure 2). The data from our study, the MOMS [2], and the International Fetoscopic Neural Tube Defect Repair Consortium (IFNTDRC) [23] are summarized and compared in Table 3 and Table 4.

In our cohort, 12 women were nulliparous (31%), and 10 had previous uterine surgery (26%), mainly cesarean section. Mean body mass index (BMI) was 27.4 kg/ m^2^ (range 18–42.4). At surgery, mean maternal age was 33 years (range 18–41) and mean gestational age at surgery was 26 weeks of gestation (range 24.4–28.1).

Most of the cases were myelomeningoceles; 6 fetuses had myeloschisis. All lesions were lumbar and sacral, starting at level L1-L2 (n = 12), L3-L4 (n = 20), and L5-S1 (n = 6). The average gestational age at surgery was 26.0 (24.4–28.1 weeks of gestation). The mean skin-to-skin time for the procedure was 253 min (120–420 min), the fetoscopic repair time was 184 min (55–360 min), and average insufflation time was 226 min (90–400 min).

In four cases surgery was abandoned: in two cases, because of difficulties in insufflating the uterus. In two other cases surgery were stopped because repeated attempts at fetal posturing were unsuccessful.

A total of 34 cases were completed successfully and ended with a live birth. Two neonates died shortly after delivery, one due to prematurity and the other one due to persistent pulmonary hypertension. Major maternal complications were uncommon. We encountered one early postoperative placental abruption after abandoning the procedure due to difficulties in positioning the fetus; it resulted in an intrauterine demise. Two abruptions occurred unrelated to the prenatal intervention at delivery, prompting cesarean sections that resulted in live births. We had one case of early postoperative chorioamnionitis and one case of amniotic fluid embolism that resolved after the cesarean section was performed. Our first patient that had five cesarean sections in total, experienced a hemorrhage 10 days after delivery that ended with a hysterectomy. Most mothers were discharged from hospital about one week after the procedure. We used patches in the first four patients. In six cases where the lesion was too big, we either added a small patch to a skin-to-skin closure or covered the lesion entirely with a patch. A total of 24 patients had the skin-to-skin closure technique without any patches used. From patient 16 onwards we added a myofascial flap, but it was only feasible in 14 operations (Table 2). The skin-to-skin technique allowed faster healing of the wound. Also, fluid leakage was more often observed in cases of patch placement.

The average gestational age at delivery was 32 weeks of gestation (26.1–37.5 weeks of gestation). The average weight of the babies delivered was 1870 g (1070–3350 g). In 90% of the cases, we experienced fluid leakage before 37 weeks of gestation. From the patients that had the one year follow-up only 13 babies out of 31 (41.9%) needed a ventriculoperitoneal shunt (Table 4). The criteria for shunting were similar to those of MOMS where we had to have a bulging fontanelle or sunsetting eyes or split sutures and at least one of the following (head circumference > 95 percentile or marked syringomyelia with ventriculomegaly or ventriculomegaly and symptoms for Chiari malformation or persistent cerebrospinal fluid leakage from the myelomeningocele wound) [2].

What is striking in our series is the difference in outcomes when the surgery is performed at or beyond 27 weeks of gestation. In these cases, the average time between surgery and delivery was 3 weeks and 3 days. In contrast, when the procedure was performed before 27 weeks of gestation, it took an average of 6 weeks and 3 days to delivery.

In this study, maternal age averaged 33 years, which was generally higher compared to the other studies, although the differences were not statistically significant. The prevalence of nulliparity and differences in body mass index across the studies were similarly not statistically significant, indicating a general uniformity in these characteristics across the cohorts. However, racial distribution differed significantly, with 100% of participants in this study identifying as White compared to a significantly lower percentage in IFNTDRC, indicating notable demographic variations between the studies [23]. The body mass index was similar across all studies, with no statistically significant differences. Previous uterine surgery rates were higher in this study compared to MOMS, but this difference was not statistically significant [2].

In the analysis of presurgical findings, the type of lesion, myeloschisis, was present in 15.8% of cases in this study, which was significantly less frequent compared to 31.3% in IFNTDRC (*p* = 0.07), suggesting a notable variance in the prevalence of this specific condition between the cohorts [23]. Cervical length before surgery also differed significantly, with this study showing a shorter average length (35 ± 6 mm) compared to MOMS (38.9 ± 7.3 mm, *p* = 0.01) and IFNTDRC (37 ± 6 mm, *p* = 0.02) [2,23]. The presence of an anterior placenta was statistically different only when compared with IFNTDRC (*p* = 0.033), not with MOMS [2,23]. Regarding surgical details, there were no statistically significant differences in the gestational age at surgery between this study and either MOMS (*p* = 0.78) or IFNTDRC (*p* = 1.00) [2,23]. However, the duration of surgery displayed significant differences, with this study having a longer average duration (236 min, range 80–420 min) compared to IFNTDRC (204 min, range 72–458 min, *p* = 0.02), suggesting differences in surgical management and procedure length [23].

In this study, the maternal outcomes, including placental abruption, chorioamniotic membrane separation, pulmonary edema, PPROM, and blood transfusion rates, showed no statistically significant differences compared to the MOMS and IFNTDRC studies [2,23]. The gestational age at birth in this study was comparable to that reported in the MOMS, however, it was significantly lower than the mean gestational age observed in the IFNTDRC study (*p* = 0.04) [2,23]. Delivery at less than 30 weeks and delivery at or beyond 37 weeks both showed differences approaching statistical significance when compared to IFNTDRC (*p* = 0.043 and *p* = 0.022, respectively) [23]. Cesarean delivery rates were notably different from IFNTDRC (*p* < 0.01) [23].

Significant differences were found in birth weight, with this study reporting a lower average birth weight compared to MOMS and IFNTDRC (*p* < 0.001 for both) [2,23]. The length of stay in the neonatal intensive care unit also differed significantly from the IFNTDRC (*p* < 0.01), indicating potentially more severe neonatal conditions in the latter group [23]. Outcomes such as perinatal death, respiratory distress syndrome, retinopathy, necrotizing enterocolitis, and periventricular leukomalacia showed no significant differences across the studies, suggesting consistent neonatal care outcomes despite different maternal and delivery conditions. Motor function outcomes relative to the lesion’s anatomic level and the incidence of hydrocephalus requiring ventriculoperitoneal shunt placement at 12 months were similarly consistent across the studies.

## 4. Discussion

The clinical implementation of percutaneous minimally invasive fetoscopic coverage of SBA was successfully achieved at our center. This accomplishment allows us to offer minimally invasive prenatal treatment to patients who are hesitant to undergo the significantly more invasive open fetal surgical approach. In our retrospective study, we analyzed perioperative, maternal, fetal, delivery, and neonatal outcomes.

One of the most important complications to monitor in these types of procedures is maternal pulmonary edema. This adverse event did not occur in any of our patients who underwent the fetoscopic approach. This may indicate that monitoring of extravascular lung water, administration of lower dosages of volatile anesthetics, and restriction of intravenous fluids to maintenance levels seem effective in reducing the occurrence of this complication [16,17,18].

As uterine blood flow is directly proportional to maternal blood pressure, it is easily understandable that significant decreases in fetoplacental and uterine blood flows have been observed during open fetal surgery in humans and during animal studies in sheep. This is because higher dosages of drugs are required to achieve sufficient maternal anesthesia and uterine relaxation [7,24,25]. The tendency toward significant intraoperative maternal hypotension to minimize maternal administration of catecholamines and intravenous fluid—as they may impair fetal hemodynamics or result in maternal pulmonary edema—may explain why fetal bradycardia during repair and postnatal periventricular leucomalacia have been observed in prenatally operated survivors in the MOMS trial and after open fetal surgery for other conditions [2,6,12,24].

Fetal surgery can be complicated by the separation of the amniotic membranes, which is a risk factor for premature rupture of the amniotic membranes [26]. In our study this complication was observed in only eight cases (21%) probably due to the use of the T-fasteners that held the amniotic membranes and uterine wall together.

In the context of performing this type of surgery, the mother’s functioning and quality of life during the remainder of the pregnancy is highly significant. Hospitalization and prolonged administration of analgesic or tocolytic agents are usually not required after fetoscopic surgery. Most patients can be discharged from the hospital within less than a week post-surgery and can remain in their communities until amniotic fluid leakage occurs or until elective readmission to our center for delivery.

Preterm delivery continues to be an important consequence for all fetoscopic procedures. Transvaginal amniotic fluid leakage for now still occurs in 90% of patients. Fortunately, leakage is prone to expectant management and, when adequately monitored for early signs of infection, can be considered a milder obstetrical complication when compared to placental abruption, any degree of uterine dehiscence, or the legacy of complications left to future pregnancies when open fetal surgery is performed. Furthermore, sutures can be used to secure the membranes at the trocar insertion sites, which should aid in reducing the incidence of leakage and prolonging gestation for several more weeks [27]. Laparotomy-assisted fetoscopic repair—a hybrid approach combining benefits of both, open and fetoscopic surgery—could potentially be a safe alternative for fetuses beyond 27 weeks of gestation. According to the literature, the hybrid technique could result in a reduced preterm birth rate and an increased gestational age at delivery compared to percutaneous fetoscopic surgery, with similar fetal outcomes [5]. However, as both approaches are novel and continue to evolve, ongoing research should be aimed at clarifying their relative benefits.

The most serious complications in our cohort occurred when the procedure was performed beyond 27 weeks of gestation. We observed early postoperative chorioamnionitis followed by fetal demise from prematurity in one case, placental abruption followed by fetal demise in another patient, and a neonatal death at 33 weeks of gestation from persistent pulmonary hypertension. For the above reasons, we find it preferable to operate on fetuses at earlier gestational ages.

A recent study on the school age functional outcomes of children that were operated upon prenatally in the MOMS trial compared adaptive behavior and other outcomes at school age (5.9–10.3 years) between prenatal versus postnatal surgery groups [28]. They did not show any difference between the groups in overall adaptive behavior (the Vineland composite score was 89.0 ± 9.6 in the prenatal group versus 87.5 ± 12.0 in the postnatal group). So, without any decrease in the overall adaptive behavior, the prenatal group gained improved mobility and independent functioning and fewer surgeries for shunt placement. In this study the prenatal group walked independently more often (29% vs. 11%; *p* = 0.06), had lower rates of hindbrain herniation (60% vs. 87%; *p* < 0.001), fewer shunts placed for hydrocephalus (49% vs. 85%; *p*< 0.001) and, among those with shunts, fewer shunt revisions (47% vs. 70%; *p* = 0.02) [28].

Moreover, the effect of the prenatal surgery on fetal and postnatal growth was analyzed, showing no difference in the growth parameters of babies exposed to surgeries in utero [29]. These results demonstrate that CO_2_ exposure during the operation is most probably safe.

The main problem of the minimally invasive fetoscopic approach, compared to the open approach, is the longer learning curve. This fact is due to the complex nature of the fetoscopic approaches and explains the higher number of surgical difficulties and complications in our early experience.

As published before, the number of cases needed to reach competency is 35 for standard hysterotomy, ≥57 cases for two to three layer mini-hysterotomy, and ≥ 56 cases for two-layer percutaneous fetoscopy. As for the single layer fetoscopy, however, competency most probably is reached at 82 cases [30].

With the growing experience in our cohort, comparison of the various minimally invasive closure techniques will become important. By design, the patch approach is the least invasive approach for the underlying neural tissues, whereas the skin-to-skin approach heals more quickly and achieves a watertight closure with less surgical experience. An additional enhancement of the closure site by a myofascial flap may provide another layer of protection. Yet the various multilayer closures by open and fetoscopic approaches exert more downward pressure on the neural cord which may result in less preservation of neurological function as well as a tendency to more tethering [19].

Given these considerations, we conducted a comparative analysis of our outcomes with the findings from the two largest studies in this field, MOMS and IFNTDRC. Our initial findings were comparable in terms of perioperative outcomes and the incidence of fetal, maternal, and neonatal complications [2,23].

Finally, it will be the analysis of the neurological outcomes of the prenatally operated children regarding the motor and sensory functions of their legs, distal bowel, and bladder, as well as the function of their anal and bladder sphincter mechanisms that will decide in the not-too-distant future which method will be the preferred one.

To sum up, serious, fetal complications occurred only in cases when operated beyond 27 weeks of gestation and no fetal deaths were directly correlated to the procedure. When comparing minimally invasive fetoscopic surgery to open fetal surgery, the former requires lower doses of anesthetics, which is beneficial to both mother and the fetus. Even though transvaginal amniotic fluid leakage was observed in most of the cases, it was not associated with any severe complications, such as placental abruption or uterine dehiscence, observed in open fetal surgery. The above findings suggest that the fetoscopic approach should be taken under consideration in fetuses diagnosed with SBA, due to its promising safety profile.

## 5. Limitations

The presented study has several limitations. First, the sample size was small. This study aimed to describe the first fetoscopic surgeries for spina bifida aperta in Poland and therefore the number of patients was limited to 34 subjects. As a consequence, the results should be considered as preliminary and interpreted with caution. Because of the small sample size it was not possible to analyze outcomes based on the closure techniques which evolved during the study. Further research involving larger cohorts is necessary to validate these findings and support the hypothesis. Second, the follow-up period was short. Assessment of long-term outcomes, especially after more than 12 months, could provide more comprehensive insights into the efficacy and safety of the procedure.

## 6. Conclusions

The findings of our study indicate that minimally invasive surgery for spina bifida aperta (SBA) can be effectively implemented following a structured and tailored training curriculum. Nevertheless, further prospective research is warranted to evaluate both maternal and neonatal outcomes in greater detail. Importantly, the fetoscopic approach allows for intraoperative assessment of different techniques of neural tube defect closure, offering potential for procedural optimization. Our data also demonstrate that preterm delivery is a common outcome; however, in the majority of cases, it occurred beyond 30 weeks of gestation. At this gestational age, severe complications related to prematurity are relatively infrequent. Additionally, this study confirms that fetoscopic repair of SBA is a safe and efficacious method. It can be reproduced with similar effects in different centers and is non-inferior to classical open surgery. Nonetheless, continued observation and long-term follow-up are essential to fully assess the safety and efficacy of the procedure.

## Figures and Tables

**Table 1:** Inclusion and exclusion criteria.

Inclusion Criteria	Exclusion Criteria
Maternal age ≥ 18 years Defect located between T1 and S1 Chiari II malformation Singleton pregnancy No other major abnormality Normal karyotype Gestational age at surgery 24–28 weeks	Multiple gestation Placenta previa Positive serology for HIV or hepatitis B Short cervix (≤20 mm) Major kyphosis > 30 degrees

**Table 2:** Evolution of surgical technique.

4 Patients	16 Patients	14 Patients
Use of patch to cover the skin, no myofascial flap [3]	Skin-to-skin closure technique (with the addition of patches in cases where the lesion was too big) No myofascial flap	Myofascial flap Skin-to-skin closure technique (with the addition of patches in cases where the lesion was too big) [20]

**Table 3:** Maternal characteristics, presurgical findings and surgical details. Comparison between this study, the multicenter randomized controlled trial Management of Myelomeningocele Study (MOMS), and the International Fetoscopic Neural Tube Defect Repair Consortium (IFNTDRC).

Characteristic	This Study (N = 38)	MOMS (N = 78)	IFNTDRC (N = 300)	*p*-Value This Study vs. MOMS	*p*-Value This Study vs. IFNTDRC
**Maternal characteristics**					
Maternal age (y)	33 (18–41)	29.3 ± 5.3	30.4 (16–45)	**0.980**	**0.990**
	[31.8 ± 6.4]				
Nulliparity	12 (31.6)	33 (42.3)	131 (43.7)	**0.363**	**0.213**
Racial origin:					
White	38 (100)	73 (93.6)	109 (36.3)	-	-
Asian	-	-	4 (1.3)		
Black	-	1 (1.3)	5 (1.7)		
Mixed	-	-	103 (34.3)		
Body mass index (kg/m^2^)	27.4 (18–42.4)	26.2 ± 3.7	26.1 (18–42)	**0.980**	**0.990**
		[26.7 ± 2.9]			
Previous uterine surgery	10 (26)	11 (14)	-	**0.** **178**	**-**
**Presurgical findings**				**-**	**0.07** **0**
Type of lesion: myelschisis	6 (15.8)	-	94 (31.3)	**0.154**	0.025 *
Anatomic level of lesion:					
Thoracic	-	4 (5.1)	15 (5)		
L1-L2	12 (31.6)	21 (26.9)	52 (17.3)		
L3-L4	20 (52.6)	30 (38.5)	133 (44.3)		
L5-S1	6 (15.8)	23 (29.5)	100 (33.3)		
Mean ventricular width of the largest ventricle (mm)	12 (6–17)	-	12 (5.6–31.5)	-	-
Anterior placenta	10 (26)	36 (46.2)	138 (46)	**0.167**	0.033 *
Cervical length before surgery (mm)	35 ± 6	38.9 ± 7.3	37 ± 6	0.010 *	0.020 *
**Surgical details**					
Gestational age at surgery in weeks	26 (24.4–28.1)	23.6 ± 1.4	25.9 (22.7–31.6)	**0.78** **0**	**1** **.000**
		[25.8 ± 0.1]			0.020 *
Duration of surgery- Skin-to-skin (min)	236 (80–420)	-	204 (72–458)		
		[221.0 ± 68.1]			

Values are expressed as mean standard deviation, minimum and maximum values, and number (percentage), as appropriate. Statistically significant values were marked with a “*”

**Table 4:** Maternal, fetal, and neonatal outcomes. Comparison between this study, the multicenter randomized controlled trial Management of Myelomeningocele Study (MOMS), and the International Fetoscopic Neural Tube Defect Repair Consortium (IFNTDRC).

Characteristic	This Study (N = 38)	MOMS (N = 78)	IFNTDRC (N = 300)	*p*-Value, This Study vs. MOMS	*p*-Value, This Study vs. IFNTDRC
**Maternal outcome**					
Placental abruption	3 (7.9)	5 (6.4)	25 (8.9)	**0.999**	**0.999**
Chorioamniotic membrane separation	15 (39.5)	20 (25.6)	72 (37.9)	**0.190**	**0.064**
Pulmonary edema	1 (2.6)	5 (6.4)	15 (5)	**0.678**	**0.800**
PPROM (weeks)	32 (84)	36 (46.2)	153 (54.6)	**0.210**	**0.350**
Blood transfusion	1 (2.6)	7 (9)	9 (3)	**0.364**	**0.990**
**Fetal or neonatal outcome**					
Gestational age at birth (weeks)	32 (26.1–37.5)	34.1 ± 3.1	34.3 ± 3.6	**0.990**	0.040 *
	[32.0 ± 2.6]				
Delivery at ≥37 wk	3 (7.9)	16 (20.5)	79 (28.2)	**0.145**	0.022 *
Delivery at ≤ 30 wk	10 (26.3)	10 (12.8)	38 (13.6)	**0.123**	0.043 *
Cesarean delivery	37 (97)	78 (100)	192:280 (68.6)	**0.990**	<0.010 *
Birthweight (grams)	1870 (1070–3350)	2383 ± 688	2270 (810–4435)	<0.001 *	<0.001 *
		[1907.90 ± 120.0]		**–**	<0.010 *
Length of stay in NICU (days)	11 (0–75)	–	17 (0–253)	**0.401**	**0.284**
		[25.5 ± 27.6]		**0.678**	**0.999**
Perinatal death	3 (7.9)	2 (3)	9 (3.2)		
Respiratory distress syndrome	9/34 (26.4)	16/77 (20.8)	40/159 (25.2)	**0.668**	**0.680**
Retinopathy	1/34 (2.94)	0 (0.0)	16/250 (6.4)	**0.999**	**0.602**
Necrotizing enterocolitis	0 (0.0)	1/77 (1.3)	8/273 (2.9)	**0.999**	**0.999**
Periventricular leukomalacia	1/31 (3.2)	4/77 (5.2)	8/258 (3.1)	**0.464**	**0.954**
**Motor function compared with the upper anatomic level of the lesion**				**0.868**	**0.373**
≥2 levels better	11/27 (40.7)	20/62 (32.3)	98/257 (38.1)	**0.393**	**0.890**
1 level better	4/27 (14.8)	7/62 (11.3)	63/257 (24.5)	**0.999**	**0.979**
Same	6/27 (22.2)	14/62 (22.6)	49/257 (19.1)	**0.352**	**0.875**
1 level worse	4/27 (14.8)	13/62 (21.0)	35/257 (13.6)		
≥2 levels worse	2/27 (7.4)	8/62 (12.9)	12/257 (4.7)	**0.990**	**0.990**
**Outcomes at 12 months**					
Hydrocephalus treated with ventriculoperitoneal shunt	13/31 (41.9)	31/76 (40.8)	88/201 (43.8)		
placement					

Values are expressed as mean standard deviation, minimum and maximum values, and number (percentage), as appropriate. Statistically significant values were marked with a “*”

## Data Availability

All data generated or analyzed during this study are included in this article. Further enquiries can be directed to the corresponding author.

## Abbreviations

The following abbreviations are used in this manuscript:

MOMS	The multicenter randomized controlled trial Management of Myelomeningocele Study
SBA	spina bifida aperta
PROM	premature rupture of membranes
STROBE	Strengthening the Reporting of Observational Studies in Epidemiology
DZFT	German Center for Fetal Surgery & Minimally Invasive Surgery
HELLP	hemolysis, elevated liver enzymes, low platelets
PE	preeclampsia
PPROM	preterm premature rupture of membranes
IFNTDRC	International Fetoscopic Neural Tube Defect Repair Consortium

## References

[1] Adzick N.S. (2010). Fetal myelomeningocele: Natural history, pathophysiology, and in-utero intervention. Seminars in Fetal and Neonatal Medicine.

[2] Adzick N.S., Thom E.A., Spong C.Y., Brock J.W., Burrows P.K., Johnson M.P., Howell L.J., Farrell J.A., Dabrowiak M.E., Sutton L.N. (2011). A randomized trial of prenatal versus postnatal repair of myelomeningocele. N. Engl. J. Med..

[3] Kohl T., Hering R., Heep A., Schaller C., Meyer B., Greive C., Bizjak G., Buller T., Van de Vondel P., Gogarten W. (2006). Percutaneous fetoscopic patch coverage of spina bifida aperta in the human—Early clinical experience and potential. Fetal Diagn. Ther..

[4] Diehl D., Belke F., Kohl T., Axt-Fliedner R., Degenhardt J., Khaleeva A., Oehmke F., Faas D., Ehrhardt H., Kolodziej M. (2021). Fully percutaneous fetoscopic repair of myelomeningocele: 30-month follow-up data. Ultrasound Obstet. Gynecol..

[5] Sanz Cortes M., Torres P., Yepez M., Guimaraes C., Zarutskie A., Shetty A., Hsiao A., Pyarali M., Davila I., Espinoza J. (2020). Comparison of brain microstructure after prenatal spina bifida repair by either laparotomy-assisted fetoscopic or open approach. Ultrasound Obstet. Gynecol..

[6] Kosinski P., Samaha R.B.B., Lipa M., Wielgos M., Kohl T. (2018). Contemporary management of prenatally diagnosed spina bifida aperta—An update. Ginekol. Pol..

[7] Pedreira D.A., Reece E.A., Chmait R.H., Kontopoulos E.V., Quintero R.A. (2016). Fetoscopic repair of spina bifida: Safer and better?. Ultrasound Obstet. Gynecol..

[8] Kabagambe S.K., Jensen G.W., Chen Y.J., Vanover M.A., Farmer D.L. (2018). Fetal Surgery for Myelomeningocele: A Systematic Review and Meta-Analysis of Outcomes in Fetoscopic versus Open Repair. Fetal Diagn. Ther..

[9] Kohl T. (2014). Percutaneous minimally invasive fetoscopic surgery for spina bifida aperta. Part I: Surgical technique and perioperative outcome. Ultrasound Obstet. Gynecol..

[10] Degenhardt J., Schürg R., Winarno A., Oehmke F., Khaleeva A., Kawecki A., Enzensberger C., Tinneberg H.R., Faas D., Ehrhardt H. (2014). Percutaneous minimal-access fetoscopic surgery for spina bifida aperta. Part II: Maternal management and outcome. Ultrasound Obstet. Gynecol..

[11] Kohl T. (2020). Minimally invasive fetoscopic surgery for spina bifida aperta: Learning and doing. Ultrasound Obstet. Gynecol..

[12] Belfort M.A., Whitehead W.E., Shamshirsaz A.A., Bateni Z.H., Olutoye O.O., Olutoye O.A., Mann D.G., Espinoza J., Williams E., Lee T.C. (2017). Fetoscopic Open Neural Tube Defect Repair: Development and Refinement of a Two-Port, Carbon Dioxide Insufflation Technique. Obstet. Gynecol..

[13] Chmait R.H., Monson M.A., Pham H.Q., Chu J.K., Van Speybroeck A., Chon A.H., Kontopoulos E.V., Quintero R.A. (2022). Percutaneous/mini-laparotomy fetoscopic repair of open spina bifida: A novel surgical technique. Am. J. Obstet. Gynecol..

[14] Zamłyński J., Olejek A., Bohosiewicz J., Bodzek P., Mańka G., Grettka K., Paliga M., Gajewska A. (2007). Perinatal results of intrauterine open fetal surgery of fetuses diagnosed with myelomeningocoele—The clinical report of ten cases. Ginekol. Pol..

[15] Von Elm E., Altman D.G., Egger M., Pocock S.J., Gøtzsche P.C., Vandenbroucke J.P., Strobe Initiative (2007). The Strengthening the Reporting of Observational Studies in Epidemiology (STROBE) statement: Guidelines for reporting observational studies. Lancet.

[16] Schneck E., Koch C., Arens C., Schürg R., Zajonz T., Khaleeva A., Kohl T., Weigand M.A., Sander M. (2017). Geburtshilfe: Anästhesie bei fetaler Chirurgie. Anasthesiol Intensiv. Notfallmed Schmerzther..

[17] Arens C., Koch C., Veit M., Greenberg R.S., Lichtenstern C., Weigand M.A., Khaleeva A., Schuerg R., Kohl T. (2017). Anesthetic Management for Percutaneous Minimally Invasive Fetoscopic Surgery of Spina Bifida Aperta: A Retrospective, Descriptive Report of Clinical Experience. Anesth. Analg..

[18] Hering R., Hoeft A., Putensen C., Tchatcheva K., Stressig R., Gembruch U., Kohl T. (2009). Maternal haemodynamics and lung water content during percutaneous fetoscopic interventions under general anaesthesia. Br. J. Anaesth..

[19] Kohl T. (2022). Lifesaving Treatments for the Tiniest Patients-A Narrative Description of Old and New Minimally Invasive Approaches in the Arena of Fetal Surgery. Children.

[20] Lapa Pedreira D.A., Acacio G.L., Gonçalves R.T., Sá R.A.M., Brandt R.A., Chmait R.H., Kontopoulos E.V., Quintero R.A. (2018). Percutaneous fetoscopic closure of large open spina bifida using a bilaminar skin substitute. Ultrasound Obstet. Gynecol..

[21] Pedreira D.A., Zanon N., Nishikuni K., Moreira de Sá R.A., Acacio G.L., Chmait R.H., Kontopoulos E.V., Quintero R.A. (2016). Endoscopic surgery for the antenatal treatment of myelomeningocele: The CECAM trial. Am. J. Obstet. Gynecol..

[22] Lapa D.A. (2019). Endoscopic fetal surgery for neural tube defects. Best Pract. Res. Clin. Obstet. Gynaecol..

[23] Cortes M.S., Chmait R.H., Lapa D.A., Belfort M.A., Carreras E., Miller J.L., Samaha R.B.B., Gonzalez G.S., Gielchinsky Y., Yamamoto M. (2021). Experience of 300 cases of prenatal fetoscopic open spina bifida repair: Report of the International Fetoscopic Neural Tube Defect Repair Consortium. Am. J. Obstet. Gynecol..

[24] Kohl T., Tchatcheva K., Weinbach J., Hering R., Kozlowski P., Stressig R., Gembruch U. (2010). Partial amniotic carbon dioxide insufflation (PACI) during minimally invasive fetoscopic surgery: Early clinical experience in humans. Surg. Endosc..

[25] Kohl T., Tchatcheva K., Merz W., Wartenberg H.C., Heep A., Müller A., Franz A., Stressig R., Willinek W., Gembruch U. (2009). Percutaneous fetoscopic patch closure of human spina bifida aperta: Advances in fetal surgical techniques may obviate the need for early postnatal neurosurgical intervention. Surg. Endosc..

[26] Amberg B.J., Hodges R.J., Rodgers K.A., Crossley K.J., Hooper S.B., DeKoninck P.L.J. (2021). Why Do the Fetal Membranes Rupture Early after Fetoscopy? A Review. Fetal Diagn. Ther..

[27] Keil C., Köhler S., Sass B., Schulze M., Kalmus G., Belfort M., Schmitt N., Diehl D., King A., Groß S. (2023). Implementation and Assessment of a Laparotomy-Assisted Three-Port Fetoscopic Spina Bifida Repair Program. J. Clin. Med..

[28] Houtrow A.J., Thom E.A., Fletcher J.M., Burrows P.K., Adzick N.S., Thomas N.H., Brock J.W., Cooper T., Lee H., Bilaniuk L. (2020). Prenatal Repair of Myelomeningocele and School-age Functional Outcomes. Pediatrics.

[29] Sanz Cortes M., Davila I., Torres P., Yepez M., Lee W., Guimaraes C.V., Sangi-Haghpeykar H., Whitehead W.E., Castillo J., Nassr A.A. (2019). Does fetoscopic or open repair for spina bifida affect fetal and postnatal growth?. Ultrasound Obstet. Gynecol..

[30] Joyeux L., De Bie F., Danzer E., Russo F.M., Javaux A., Peralta C.F.A., De Salles A.A.F., Pastuszka A., Olejek A., Van Mieghem T. (2020). Learning curves of open and endoscopic fetal spina bifida closure: Systematic review and meta-analysis. Ultrasound Obstet. Gynecol..

